# A Single Centre Retrospective Evaluation of Laparoscopic Rectal Resection with TME for Rectal Cancer: 5-Year Cancer-Specific Survival

**DOI:** 10.1155/2011/473614

**Published:** 2011-10-18

**Authors:** Raoul Quarati, Massimo Summa, Fabio Priora, Valeria Maglione, Ferruccio Ravazzoni, Luca Matteo Lenti, Graziella Marino, Federica Grosso, Giuseppe Spinoglio

**Affiliations:** ^1^Department of Surgery, SS. Antonio e Biagio National Hospital, Alessandria, Italy; ^2^Oncohematologic Department, A.S.O. SS. Antonio e Biagio e C. Arrigo, Via Venezia 16, 15100 Alessandria, Italy

## Abstract

Laparoscopic colon resection has established its role as a minimally invasive approach to colorectal diseases. Better long-term survival rate is suggested to be achievable with this approach in colon cancer patients, whereas some doubts were raised about its safety in rectal cancer. Here we report on our single centre experience of rectal laparoscopic resections for cancer focusing on short- and long-term oncological outcomes. In the last 13 years, 248 patients underwent minimally invasive approach for rectal cancer at our centre. We focused on 99 stage I, II, and III patients with a minimum follow-up period of 5 years. Of them 43 had a middle and 56 lower rectal tumor. Laparoscopic anterior rectal resection was performed in 71 patients whereas laparoscopic abdomino-perineal resection in 28. The overall mortality rate was 1%; the overall morbidity rate was 29%. The 5-year disease-free survival rate was 69.7%, The 5-year overall survival rate was 78.8%.

## 1. Introduction

Laparoscopic colon resection has established its role as a minimally invasive approach to colorectal diseases. 

Some doubts were raised about its feasibility and safety in rectal cancer because of concern related to thorough surgical exploration in narrow pelvis and correct total mesorectal excision with the laparoscopic technique. The risk of port site metastases had also been previously emphasised [1, 2].

No conclusive data are available in this setting, and further trials are deemed to be needed [3–6].

However, several studies demonstrated the technical feasibility and safety of laparoscopic rectal resections for cancer. Many authors showed advantages for laparoscopic colorectal surgery in terms of reduced postoperative pain, shorter postoperative ileus, and length of hospital stay [3, 7–10]. As a medium-term advantage, a reduced rate on incisional hernia should be considered [11]. A better postoperative recovery, especially for older patients, was also reported [12].

From an oncological point of view, the noninferiority of colic laparoscopic surgery was established [13]. A better long-term survival rate in colon cancer patients has been suggested in some experiences [14]. 

Here we report on our rectal cancer patient series treated with laparoscopic approach that was retrospectively analysed focusing on functional results and oncological outcomes.

## 2. Patients and Methods

Three hundred and thirty-one patients with rectal cancer underwent surgical treatment at our hospital from June 1997 to December 2010; 248 of them had minimally invasive approach (192 laparoscopic—49 fully robotic). 

We selected for this analysis 102 patients with a minimum followup of 5 years, therefore excluding 146 patients operated on after December 2005. Three more patients with UICC Stage IV disease were also excluded. Eventually we analysed 99 patients, 43 (45.7%) with a middle tumor (>8 cm from anal verge) and 56 with a low tumor (<8 cm from anal verge).

A rectal anterior resection (RAR) was performed in 71 patients (71.7%) and a laparoscopic abdominoperineal resection (APR) in 28 (28.3%). All the patients were operated on by the same surgeon (GS).

A written and detailed informed consent was obtained from each patient. Age at surgery, gender, pathological tumor stage (according to the pTNM classification), and other relevant variables were prospectively recorded for each patient in an appropriate data base implemented since the beginning of our laparoscopic activity. 

Preoperative workup included colonoscopy, contrast-enhanced CT enema, thoracic CT, magnetic resonance of the pelvis, and endorectal ultrasonography. 

After preoperative assessment 68 patients (68%) with extrarectal tumor diffusion (T3 stage) or nodal metastasis (N+) received neoadjuvant radiochemotherapy.

Obesity or previous abdominal surgery was not considered contraindications for laparoscopic surgery.

All rectal resections were carried out with inferior mesenteric vessel ligation and left flexure detachment with a medial-to-lateral approach. 

Total mesorectal excision (TME) was performed in all patients according to the Heald's principles [15].

Bowel reconstruction was performed by Knight-Griffen colorectal anastomosis. 

J-pouch was used both in coloanal hand sewn anastomosis and in mechanical anastomosis within 2 cm from the dentate line, whenever possible.

To preserve sphincter function in very low tumors, bowel reconstruction was performed after intersphincteric resection, by coloanal hand sewn anastomosis. 

A diverting loop ileostomy was performed in 53 patients undergone RAR (14 middle tumors, 39 lower tumors). In all cases a wound protector was used to extract the specimen.

The clinical parameters analysed were patient variables, operative variables, and clinical outcomes. Patient variables were age at surgery, gender, and pathological tumor stage (according to the pTNM classification) (Table 1). Operative variables included operating time and conversion rate. Clinical outcomes were surgical complications, recurrence rate, site of first recurrence, disease-free survival, and overall survival. 

Follow-up protocol included a medical examination and serum CEA determination every 3 months for two years, every 6 months for the third year, and annually thereafter. An abdominal sonography, with systematic research of liver metastasis, was performed every 6 months. Additional radiological imaging (chest X-ray, CT scan, MRI scan, etc.) was carried out if appropriate. A flexible colonoscopy was performed every year.

The cancer-specific disease-free survival rate was analysed with a minimum followup of five years: data were considered as uncensored only if the patient died as a direct result of colorectal cancer; deaths from all other causes were censored.

The Kaplan-Meier method was used to plot the survival curves, and the log-rank test was used for their comparison. 

A *P* value of less than 0.05 was set as the statistical significance level.

Pearson's chi-squared test, “*t*” test, or Fisher's exact test was used when appropriate.

Statistical analysis was performed with commercially available software (SPSS version 13.0, SPSS Inc., Chicago, Ill, USA).

## 3. Results

The age, distribution, and gender of the study population is showed in Table 1. 

Mean operating time was lower for middle rectal cancer group (mean 210 min.) than for low rectal cancer group (mean 270 min.) (*P* < 0.01).

The conversion rate was 10%, mainly due to adhesions, difficult isolation of locally advanced bulky tumors, or septic complications.

The postoperative mortality rate was 1%; there was 1 fatal complication, with postoperative death, due to multiorgan failure in systemic candidiasis.

The overall morbidity rate was 29%.

Postoperative complications included 3/71 (4.2%) cases of anastomotic leakage, 3/99 (3%) wound infections, 5/71(7%) anastomotic bleeding, 8/99 (8%) transitory urinary retention, and 2/99 (2%) small bowel obstruction (Table 2).

Patients with anastomotic leakage needed reintervention with creation of a diverting ileostomy, peritoneal lavage, and drainage.

There were no positive proximal, distal, or circumferential margins. The mean number of harvested lymph nodes was 19 (range 2–75).

After a median followup of 72 months (min 60 and maximum 146), 21 (21,2%) patients died from colorectal cancer and 27 (27,2%) had a cancer recurrence.

The most common site of recurrence was the lung, followed by the liver. There were 3 cases of local recurrence (Table 3). No cases of peritoneal seeding or portsite recurrence were reported.

**Figure 1:**
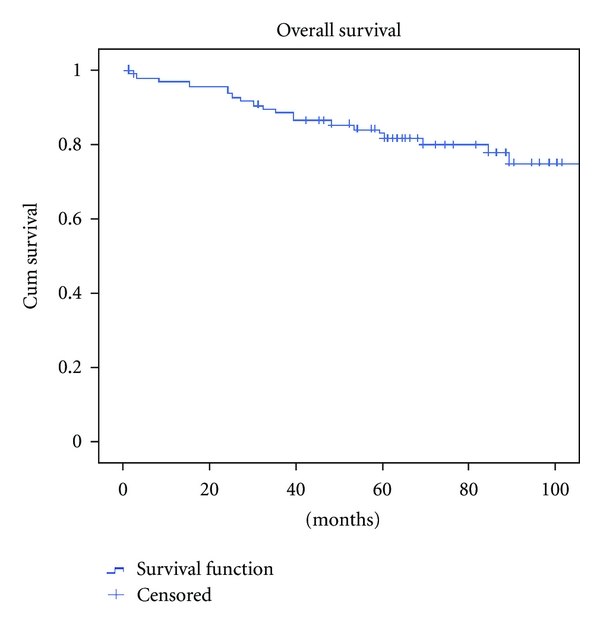
Overall survival.

**Figure 2:**
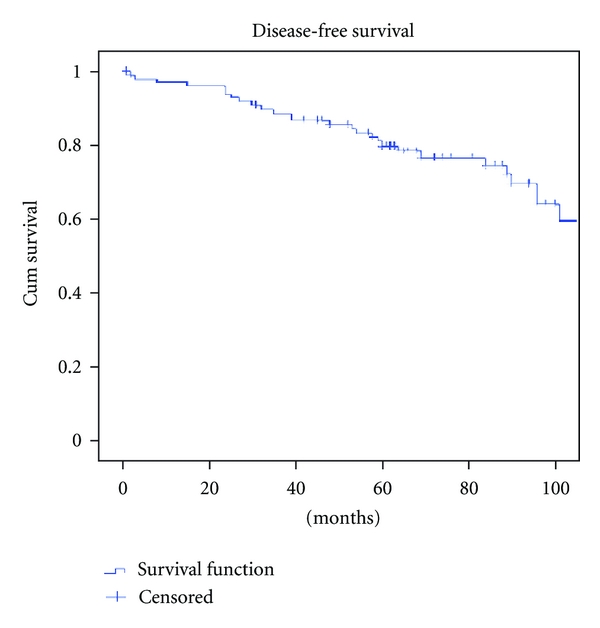
Disease-free survival.

The 5-year disease-free survival (DFS) rate was 69.7% (Figure 1). The DFS stratified per stage was 75.6%, 65.7%, and 65.2% for patients in stage I, stage II, and stage III, respectively (Figure 2). 

**Figure 3:**
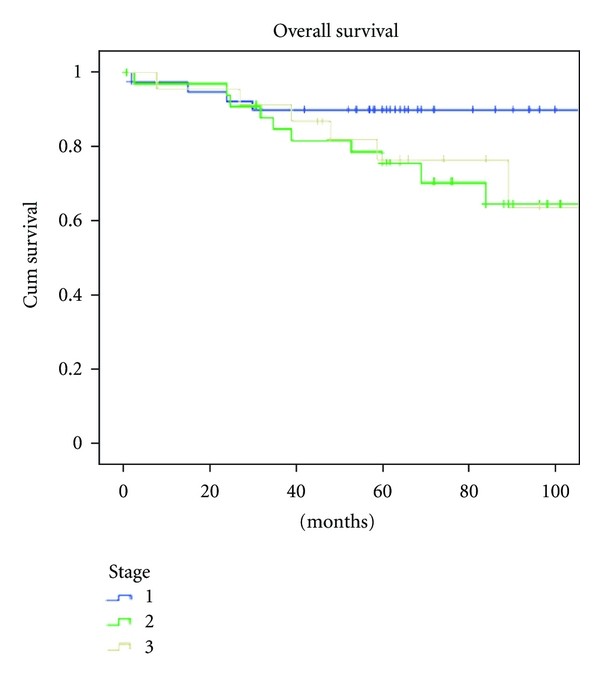
Overall survival stratified per stage.

**Figure 4:**
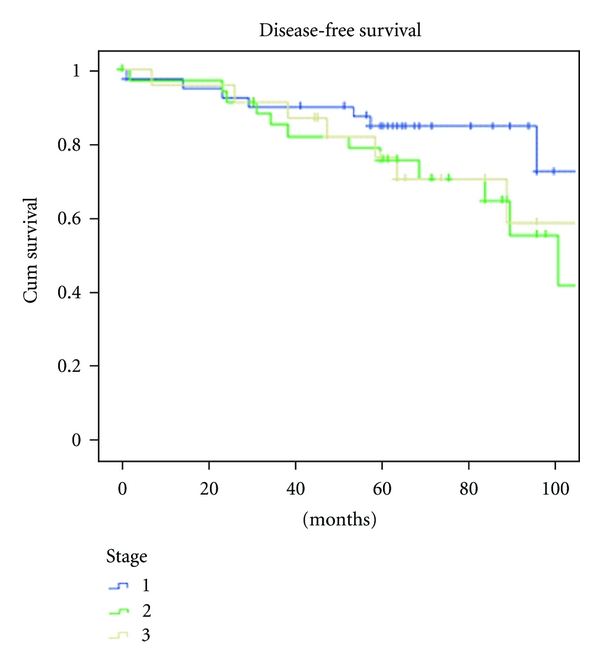
Disease-free survival stratified per stage.

The 5-year overall survival (OS) rate was 78.8% (Figure 3). The OS stratified per stage was 87.8%, 71.4%, and 73.9% for patients in stage I, stage II, stage III, respectively (Figure 4). 

## 4. Discussion and Conclusions

In this single centre series of 99 rectal cancer patients treated with minimally invasive approach the 5-year DFS rate was 69.7% and the 5-year OS rate was 78.8%, with 10% of conversion rate and 29% of overall morbidity rate. 

Though laparoscopic colon surgery has gained popularity because of its positive influence on short-term outcome, it should be kept in mind that the first aim of colorectal cancer surgery is to ensure oncological outcomes at least similar to those of open surgery. 

Rectal laparoscopic surgery is still a debated issue. The MRC-CLASICC trial in 2005 [3] had reported impaired short-term outcomes after laparoscopic anterior resection concluding that its use for rectal cancer could not be yet justified. In 2008 Kim et al. [6] reported an increasing tendency for positive circumferential margins, leak, and local recurrence in laparoscopic resection for extraperitoneal rectal cancer. The Cochrane Review [16] in 2008 concluded that laparoscopic rectal resection with TME appears to have clinically measurable short-term advantages in patients with primary rectal cancer. 

Its long-term impact on oncological endpoints awaits the results from the on-going randomized trials.

Since 1997 in our surgical department laparoscopy has been largely used and gradually replaced open surgery. We started laparoscopic rectal cancer surgery at a very early phase, and in this paper we report on long-term outcome of a large series of rectal cancer patients. Though acknowledging the overt limitation of this retrospective study, the data have been collected prospectively in an appropriate data base that was used since the beginning of our laparoscopic activity. The percentage of patients lost at followup in our series is less than 5%. Our department is a referral center for advanced laparoscopic surgery. This analysis was mainly undertaken to assess our results in rectal cancer laparoscopic surgery, being the evaluation of functional results and oncological outcomes of the primary end point. At that time there was still serious concern about possible inadequacy of tumour and lymph nodes resection and risk of cancer dissemination at the port sites. 

The strength of our study consists in the series size and in the length of followup. 

The conversion rate was superimposable to that reported in previous studies whereas the wound infection rate after laparoscopic surgery seemed lower than that previously reported for open colorectal surgery [17, 18].

From a functional point of view laparoscopic magnification allows identifying and preserving hypogastric and pelvic nerves during the IMA isolation, medium to lateral dissection, and TME. Preservation of sexual function after laparoscopic surgery is still a matter of debate. Ad hoc questionnaire on sexual activity is not often administered in clinical studies [19]. Even in our series we did not collect any information in this regard. 

In the same way we did not specifically study the urinary function but we registered in our data base any clinically relevant event including urinary tract symptoms: all but 8 patients in this series had the catheter removed within 2 days after surgery and no permanent urinary dysfunction was recorded.

The choice to perform a straight colorectal anastomosis after rectal resection was due to the favourable functional results (patient satisfaction) observed in our laparoscopic and open surgery experience and the ease of implementation of this procedure. The straight anastomosis is useful in case of narrow pelvis, obese patients, diverticular colon (contraindication to perform J-pouch), and limited colon mobilization is needed.

Cochrane meta-analysis in 2008 compared three reconstructive techniques after anterior rectal resection (straight, J-pouch, coloplasty) by analyzing 9 RCTs on straight versus J-pouch anastomosis: J-pouch seems superior for short-term functional results (within 8 months from the operation) with the same complication rate, whereas long-term functional results tend to overlap. Of note, the 9 RCTs were all been published before 2002, and none of them considered the laparoscopic approach [20].

With respect of the use of a diverting loop ileostomy, although some reports indicate that diversion does not influence the leakage rate, our results suggest that this could not be the case. In fact the lower incidence of anastomotic leakage seems to correlate with the use of diverting loop ileostomy.

In line with previous studies our data suggests that laparoscopic rectal resection provides similar oncological long-term outcomes compared to open rectal resection (DFS 52.1%–81%, OS 52,9%–75,3) [17, 21–25]. The oncological safety issues of laparoscopic approach, in terms of number of harvested lymph nodes, recurrence rate, and cancer-related survival, are all in line with those reported in Abraham's meta-analysis in 2004 [21]. 

We chose to limit our analysis to those patients with a minimum followup of 5 years. Considering that most of the cancer-related deaths occur within the first two years after surgery, such a long time frame seems to be enough to draw some initial conclusions. 

In conclusion, in an advanced laparoscopic surgical setting, laparoscopic rectal resection is feasible and seems to accomplish almost the same five-year survival and recurrence rate as open rectal resections.

Based on the already demonstrated short- and medium-term advantages of laparoscopic surgery and in light of our experience, we support this approach and think that it could deserve more extensive application. 

## Figures and Tables

**Table 1:** Patients characteristics.

Characteristic	Middle tumor (*n* = 43)	Lower tumor (*n* = 56)
Mean age (range)	71 (42–96)	70 (42–91)

Gender		
Males	25	34
Females	18	22

Stage TNM		
I	8 (17%)	5 (42%)
II	13 (50%)	24 (30%)
III	22 (33%)	27 (28%)

**Table 2:** Complications.

Complications	No. of patient (%)
Urinary retention	8 (8%)
Anastomotic bleeding	5 (7%)
Wound infection	3 (3%)
Anastomotic leakage	3 (4,2%)
Small bowel obstruction	2 (2%)

**Table 3:** Local and distant recurrence.

Site of recurrence	*N* = 27
Lung	13 (48%)
Liver	7 (25%)
Local	3 (11%)
Lymph nodes	2 (7%)
Peritoneum	1 (3%)
Brain	1 (3%)
